# A Comparative Evaluation of Enamel Wear Against Different Surface Finished Ceramics: An In Vitro Study

**DOI:** 10.7759/cureus.44689

**Published:** 2023-09-04

**Authors:** Jovin Cherian, Rahul Jayakumar, Jittin James, Vishnu Thomas, Sethu Sramadathil, Athira Kattachirakunnel Sasi

**Affiliations:** 1 Department of Prosthodontics, Al-Azhar Dental College, Thodupuzha, IND; 2 Department of Prosthodontics, Indira Gandhi Institute of Dental Sciences, Kothamangalam, IND

**Keywords:** ips empress, zirconia, polished, overglazed, autoglazed

## Abstract

Background: An essential factor in the clinical acceptability of all ceramic restorations is the degree of tooth enamel wear. Wear of human enamel has been shown to produce traumatic occlusion, loss of vertical dimension, supra eruption of opposing teeth, periodontal disintegration, and temporomandibular dysfunction. To assess and compare enamel wear against two different ceramics (IPS Empress and Zirconia) and against three different ceramic surface treatments (autoglazed, overglazed, and polished).

Methods: Zirconia and IPS Empress (Ivoclar Vivadent, Liechtenstein) ceramic samples totaled 30, of which 10 each underwent glaze, overglaze, or polishing with Diaglaze polishing paste. Using a horizontal pin on the disc machine, 60 mounted premolar tooth samples were produced and put through wear tests against ceramic discs. Prior to and following wear, the weight of the tooth samples was measured, and the results were then statistically analyzed using the student t-test, unpaired t-test, and analysis of variance (ANOVA). The qualitative data employed proportions, and the quantitative data used mean and standard deviation to express statistical information. The threshold of significance was set at 5% (α = 0.05).

Results: The results for weight loss after 25,000 cycles for ceramic surfaces that had been overglazed were just a little bit greater than those for ceramic surfaces that had been autoglazed and polished. In each of the various subgroups, it was shown that the weight loss values obtained with polished ceramic after 25,000 cycles were significantly lower than those obtained with autoglazed and overglazed ceramic surfaces (P=0.001). When comparing the results produced by the two separate primary groups, IPS Empress and Zirconia, there was no statistically significant difference between the autoglazed, overglazed, and polished groups. While statistically significant difference was seen for each subgroup of IPS and Zirconia (p ≤ 0.01), particularly Zirconia glazed (1.227) with a highly significant p-value of 0.00.

Conclusion: The findings contribute to the understanding of the potential clinical implications of different ceramic materials and surface finishes in restorative dentistry, offering valuable insights for practitioners in their treatment decisions. Further research and clinical observations may be needed to corroborate these findings and guide evidence-based practices in dental restorations.

## Introduction

Any dental restoration's primary consideration is aesthetics. The development of all-ceramic restorations is a result of the quest for better aesthetics. Due to their great aesthetic, biocompatibility, low thermal conductivity, and wear resistance characteristics, dental ceramics closely resemble genuine teeth [1]. All-ceramic restorations' potential for achieving aesthetics is undoubtedly what continues to be the key factor in people choosing them. Ceramics are now used in a number of rehabilitation operations, such as inlays, onlays, and crowns that repair the posterior occlusal surface, as a result of improved materials and cutting-edge techniques [2]. The abrasive activity of restorative materials on the opposing natural dentition has never totally been eliminated despite the continuous advancement of these materials and continues to be a clinical challenge. Based on a number of investigations by Seghi and Mange, it has been established that ceramic materials largely wear by abrasion and that this degradation is more closely related to roughness and fracture resistance than hardness values [3,4].

It has been demonstrated that the wear of human enamel can cause traumatic occlusion, loss of vertical dimension, supra eruption of opposing teeth, periodontal disintegration, and temporomandibular dysfunction. Wiley, using qualitative techniques, has clinically documented this wear and its adverse effects [5]. A variety of procedures for treating ceramic restorations have been suggested. To provide a smooth surface and reduce plaque retention, the surfaces of all dental porcelains are polished or glazed. This prevents enamel wear [6]. In a study, Krejci et al. [7] came to the conclusion that the opposing restoration's hardness, texture, and surface finish all affect how quickly enamel wears off. Several researchers have proposed fine polishing as a glazing substitute. Reglazing or repeated firing of the surface more than once needs more time and runs the risk of devitrification, which increases opacity and drains vitality.

The IPS Empress (Ivoclar Vivadent, Liechtenstein), widely utilized in dentistry, is unique due to its composition containing lithium disilicate and fluorapatite crystals. This composition confers enhanced fracture resistance, reduced antagonist tooth wear, and exceptional aesthetics. Also, they have properties that are very similar to dental structures, such as light transmission, color reproduction, and texture. As the use of these ceramic restorations in clinical practice increases, it is critical to evaluate their wear potential. Additionally, there is not enough research on zirconia's surface finishes on enamel. Utilizing ceramics that are less abrasive would improve aesthetics while reducing the deterioration of opposing teeth [8]. Therefore, the intent of this investigation is to assess and correlate how enamel wear is impacted by various ceramic surface finishes (autoglazed, overglazed, and polished).

## Materials and methods

Sixty samples of ceramic discs were split evenly into 30 samples of IPS Empress (IPS e.max CAD (Ivoclar Vivadent, Liechtenstein)) and Zirconia Y-TZP (AmannGirbach, Austria). Each of these samples was further subdivided into three groups of 10 samples each of which are glazed, overglazed, and polished. Sixty samples each of a diameter of 10mm and thickness of 1mm were fabricated. Thirty were fabricated in lithium disilicate glass ceramic and the remaining 30 in zirconium oxide ceramics.

**Figure 1:**
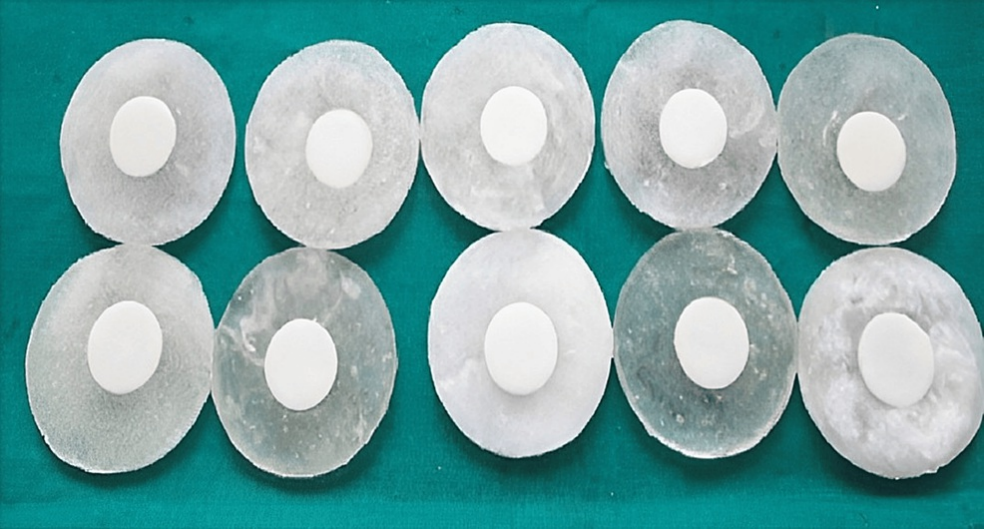
Prepared discs

Copy milling followed by sintering was used to fabricate the core, and the shape and size of the disc were standardized using a custom-made metal mold. Zirconia block (Ceramill CAD, AmannGirrbach, Germany) and Lithium disilicate glass-ceramic block (IPS e.max CAD) were subjected to milling using a computer-aided design (CAD) and computer-aided manufacturing (CAM) milling machine (Imes-icore 250 I dry, Leibolzgreben, Germany) after the images copied to CAD software. The milling was done for 40 minutes for each specimen and the milled specimens were larger in dimensions to compensate for the shrinkage during firing. The sintering was performed at a temperature range of 1250ºC to 1300ºC for 10 hours. After the specimens cooled off, they were trimmed and finished to the desired dimensions. Specimens were subjected to sandblasting (Sirio dental division sandblasting machine (Sirio Dental, Meldola, Italy)) 50 μm of granules size at 50 PSI and were used at constant pressure and ultrasonic cleaning for 180 seconds (digital ultrasonic cleaner) and layered in order to achieve a thickness of 1mm. Each of the ten samples of IPS Empress and Zirconia was autoglazed; 10 samples of IPS Empress and Zirconia were overglazed by adding an additional layer of glaze. Similarly, 10 samples of IPS Empress and Zirconia were polished with the Diaglaze polishing paste (Dentkart, India) using the Yeti lubricated impregnated felt wheels at a speed of 7500 rpm using a micromotor (Figure 1) [2,3].

The sample size for the study was figured up following the formula provided. n= [Z alpha/2. sigma/E]^2, where 'Z alpha/2' is known as the critical value, the positive 'Z' value that is at the vertical boundary for the area of 'alpha/2' in the right tail of the standard normal distribution where 'sigma' is the population standard deviation and 'n' is the sample size.

**Figure 2:**
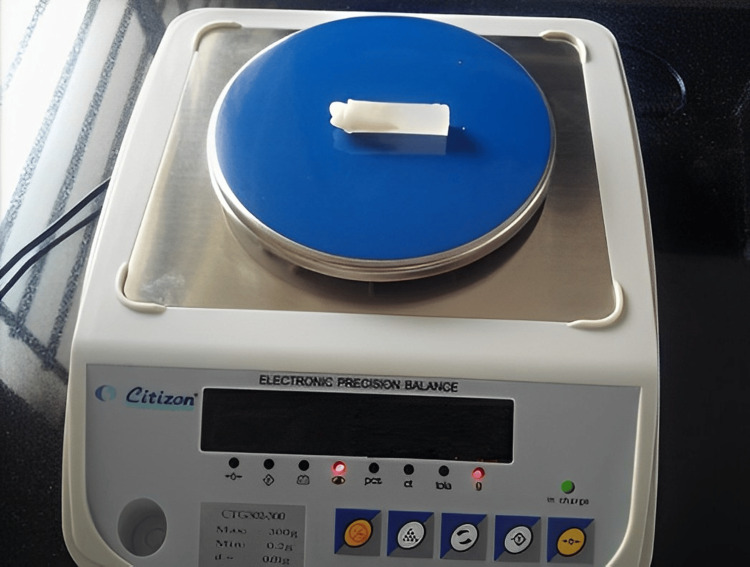
Weighing the sample

For the study, 60 freshly removed, not filled, not decayed, non-attrited upper first premolars from teen orthodontic patients were gathered, treated with 10% formalin, and kept in saline. Under a stereomicroscope, Zoom (MV-NSZ-405, Leika company, USA), the occlusal structure of each tooth was examined. Teeth with pointed cusps and appropriate morphology were chosen, affixed in blocks of self-curing acrylic resin, and arbitrarily split into six sets of 10. Before evaluation, we used a Sartorius electronic analytical balance (Sartorius AG, Germany) with a resolution of 0.0001 g to determine the mass samples. After being dried using absorbent paper, each value could be precisely determined by using this electronic machine's completely automated calibration technology and a micro weighing scale (Figure 2).

**Figure 3:**
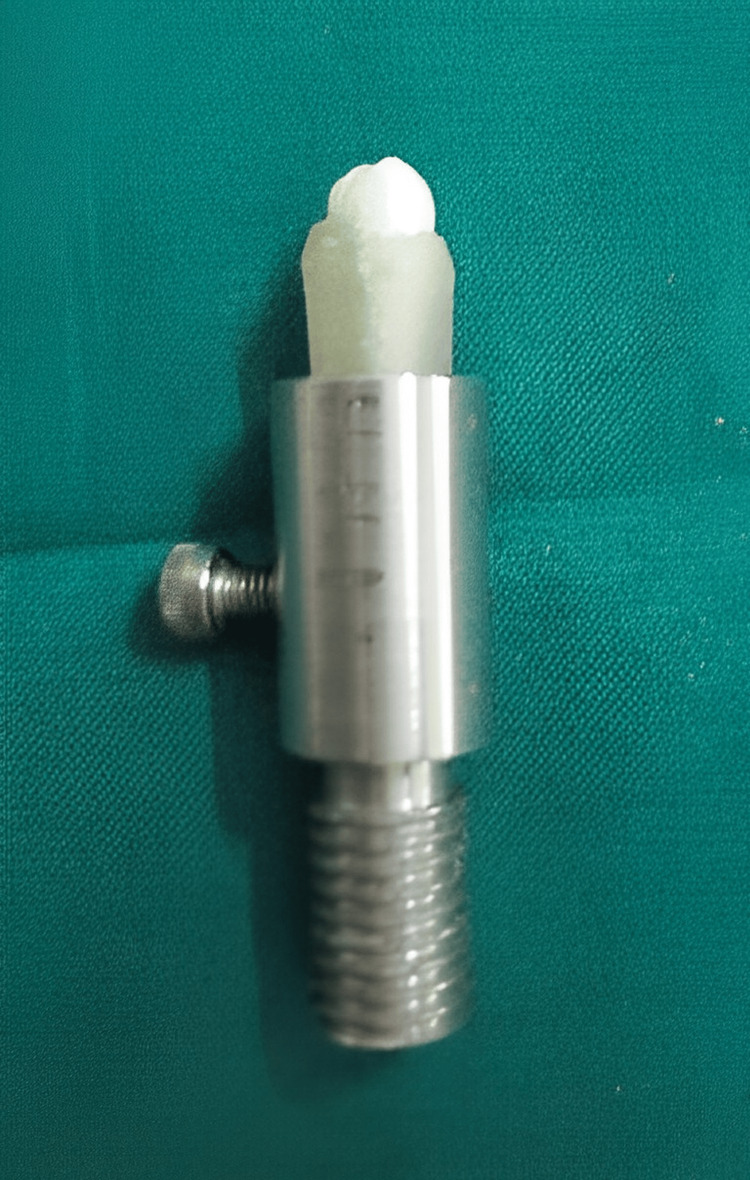
Acrylic block holder

The balance was maintained on a freestanding table, and the weights were measured with the glass doors of the balance closed to eliminate the possibility of error due to air movement. The pin-on-disc test involves pressing a pin under a known stress onto a revolving disc at a single point of contact to measure the amount of wear on both the pin and the disc. The wear volume may be calculated by either the mass reduction of the specimens or the pin shortening and disc wear track volume calculations in this test. It measures sliding friction and wear in dry, lubricated, regulated, and partial vacuum conditions. The ceramic discs were mounted in acrylic blocks which were made to the size of the holders and were positioned to the respective holders along with the samples on a two‑body wear machine (Figure 3).

**Figure 4:**
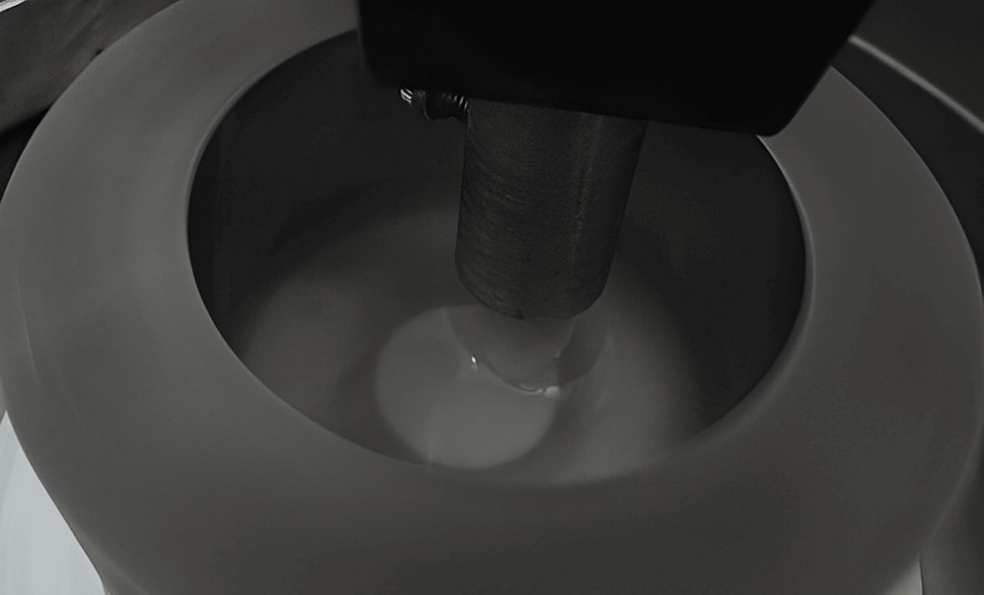
Samples exposed to wear machine

The upper and lower members (rotating wheel) held the disc and tooth accordingly. They were subjected to a steady load of 40N and sprinkled with synthetic saliva throughout the experiment. To mimic the 2D wear and tear of the mouth, the specimens were designed to glide against one another. Each pair of samples was subjected to 25,000 cycles of the wear machine and then weighed (Figure 4) [2].

The study was conducted at KLE Society’s Institute of Dental Sciences in association with the Indian Institute of Science and Smile Kraft dental lab in Banglore. Ethical Committee clearance was obtained from the Institutional Ethical Committee with letter no.: KIDS/IEC/11-2015/28. The SPSS statistical analysis program (IBM SPSS Statistics for Windows, Version 19.0: IBM Corp, Armonk, USA) was used to statistically analyze the data that was thus acquired. The threshold of significance was set at 5% (α = 0.05). For the purpose of estimating any significant differences between groups, a one-way analysis of variance (ANOVA) test was conducted. The data were compared using non-parametric testing, namely the paired sample t-test and the independent t-test. While the qualitative data employed proportions, the quantitative data used mean and standard deviation to express statistical information.

## Results

The statistical study of the mean values of enamel loss in comparison to IPS Empress and Zirconia was done. ANOVA was used to compare the means of different groups, paired sample t-tests, and independent t-tests to determine the average loss of enamel height with P < 0.05 and confidence intervals set at 95%.

Relative percentage change in weight was computed by: 100 × (baseline weight-weight during different cycles)/(baseline weight)

Table 1 lists the descriptive data for each IPS Empress and Zirconia subgroup. The aggregate mean-tested values indicate little variance. Enamel against IPS overglazed (0.24 ± 3.41) and Zirconia polished (0.15 ± 3.55) showed the largest and lowest standard deviations, accordingly.

**Table 1:** The table represents the mean value and standard deviation of the various subgroups under IPS empress and Zirconia.

Groups		Weight	Minimum	Maximum	Mean	Standard Deviation
IPS	Glazed	Before	3.1117	3.7416	3.401830	0.2215168
		After	3.1104	3.7406	3.400400	0.2217011
	Overglazed	Before	3.1172	3.8164	3.414750	0.2462018
		After	3.1152	3.8148	3.413150	0.2464772
	Polished	Before	3.3224	3.7814	3.502370	0.1768475
		After	3.3200	3.7789	3.499850	0.1768688
Zirconia	Glazed	Before	3.2266	3.8442	3.520850	0.1758881
		After	3.2250	3.8438	3.513600	0.1761130
	Overglazed	Before	3.2365	3.8133	3.432450	0.1787536
		After	3.2345	3.8091	3.430240	0.1781095
	Polished	Before	3.4018	3.8160	3.554880	0.1522072
		After	3.4010	3.8154	3.554020	0.1522396

The paired samples analysis is shown in Table 2, and it demonstrated substantial statistical significance for each subgroup of IPS and Zirconia (p ≤0.01). The means of two measures (taken before and after wearing each of the two materials) are compared using a paired sample t-test. Among them, Zirconia glazed (1.227) has the lowest value and a highly significant p-value of 0.00.

**Table 2:** The table represents the statistical analysis by paired sample t-tests *p≤0.01 statistically highly significant, **p≤0.05 statistically significant

Groups		t value	p-value
IPS	Glazed	11.62	0.00*
	Overglazed	10.05	0.00*
	Polished	13.05	0.00*
Zirconia	Glazed	1.227	0.00*
	Overglazed	8.119	0.00*
	Polished	11.27	0.00*

The differences between and within the groups of both materials (IPS and Zirconia) are shown by the one-way ANOVA values. Here, the F ratio, which is the ratio of two mean square values for IPS (0.662) and Zirconia (1.391) with a p-value (p>0.05), demonstrated the statistical insignificance (Table 3).

**Table 3:** The table represents the statistical analysis by one-way analysis of variance (ANOVA) to compare the values between and within the groups of IPS Empress and Zirconia after subjecting it to wear. DF - degree of freedom, *p≤0.01 statistically highly significant, **p≤0.05 statistically significant

Groups		Sum of Squares	DF	Mean Square	F	p-value
IPS	Between Groups	0.059	2	0.029	0.622	0.544
	Within Groups	1.271	27	0.047		
Zirconia	Between Groups	0.080	2	0.040	1.391	0.266
	Within Groups	0.773	27	0.029		

The independent sample t-test statistical analysis used to compare the values of the Zirconia and IPS Empress samples after wearing (Table 4) was determined to be non-significant. The p-values for glazed surfaces (0.22), overglazed surfaces (0.86), and polished surfaces (0.47) are all higher than 0.05.

**Table 4:** The table represents the statistical analysis by independent sample t-test to contrast values of Zirconia and IPS Empress samples after subjecting it to wear. *p≤0.01 statistically highly significant, **p≤0.05 statistically significant

After wearing	Groups	t value	p-value
Glazed	Zirconia	1.26	0.22
	IPS		
Overglazed	Zirconia	0.17	0.86
	IPS		
Polished	Zirconia	0.73	0.47
	IPS		

The results for weight loss after 25,000 cycles for ceramic surfaces that had been overglazed were just a little bit greater than those for ceramic surfaces that had been autoglazed and polished. In each of the various subgroups, it was shown that the weight loss values obtained with polished ceramic after 25,000 cycles were significantly lower than those obtained with autoglazed and overglazed ceramic surfaces (P 0.001). When comparing the results produced by the two separate primary groups, IPS Empress and Zirconia, there was no statistically significant difference.

## Discussion

Among the several ceramic systems available, pressed ceramics feature qualities including light transmission, color reproduction, and texture that are very similar to tooth structures. Zirconia is more durable than metals, resembles teeth in color, and costs less than gold. Zirconia, a crystalline dioxide of zirconium, is gaining a lot of attention as a restorative material as a result of these qualities. Due to the unequal dispersion of crystals within a glassy matrix, ceramic materials are inherently flawed in numerous ways. Enamel's strength is reduced and its wear rate is increased by additional defects introduced during ceramic production [9].

When done at a low temperature, surface glazing creates a glassy, hygienic surface and boosts the strength of the ceramic. To find finishing and polishing methods that would produce surfaces as smooth as or smoother than glazed porcelain, numerous investigations were carried out. The initial smoothness of a glazed surface was considered by some authors to be superior to a polished surface, while others found no discernible difference between the two, and still, others came to the conclusion that surface polishing may match or surpass the smoothness attained with surface glazing. Changes in the degree of enamel wear could be caused by changes in surface roughness [10-13].

In a sliding wear test, Monasky and Taylor [14] reported functional polishing of porcelain during the wear process. They discovered that the rapid rate of wear early on; gradually declined, indicating that surface roughness's impact can be self-limiting. Nevertheless, a lot of studies have not been conducted on the surface polishes of zirconia once it is subjected to occlusal adjustments. Hence, we conducted this research to check the enamel wear against two different commonly used CAD/CAM-based ceramics i.e. IPS Empress (IPS E-max) and Zirconia, and to also evaluate the wear of the different surface finishes of the two different ceramics. In this study, 60 ceramic discs were fabricated of which 30 were of IPS Empress (IPSE-max) and 30 were of Zirconia (Amann Girrbach). Ten each of the respective samples were then subjected to autoglazing, overglazing, and polished using Diaglaze diamond paste. They were disinfected in formalin and stored in saline. They were then affixed in acrylic blocks. Once the samples were prepared they were subjected to wear on the horizontal pin on the disc machine which was done in accordance with studies conducted by Mulay, Hiyasat, and Saunders [2,15].

Previous studies conducted by Seghi and Rosenstiel [3] used an apparatus that was designed to produce continuous sliding contact between enamel and ceramic discs and studies conducted by Harrison and Lewis [16] used a wear-testing machine that had modified parts such as a motor to drive the machine and digital display for monitoring contact between stud and plate specimens with water pump and heater. For this research, to replicate the oral wear cycle, the specimens were made to slide against one another. After placing specimens in holders, each pair of samples underwent 25,000 cycles of the wear machine testing, which simulates a year's worth of clinical wear under typical intraoral circumstances. To imitate oral conditions and serve as a cooler, the cusp tips and ceramic discs were placed under a steady load of 40N and sprinkled with synthetic saliva throughout the experiment. The tooth specimens were weighed before and after subjecting it to wear. Artificial saliva was prepared in the Biochemistry department of KLE Dental College. It was formulated according to the Macknight, Hane, and Whitford (1992) method [17].

In the IPS Empress and Zirconia group, it was seen that there were significant changes in the weight of the teeth within the individual subgroups. There were changes in the weight of the tooth samples when they were subjected to wear (p=0.00). The results indicate that enamel loss was significantly different (P < 0.001) depending on the surface condition (autoglazed, overglazed, and polished). It was seen that wear was maximum for the overglazed group and less for polished groups. This was in accordance with studies conducted by Elmaria et al. [6] who evaluated the wear of IPS ceramic in glazed and polished conditions and concluded that polished ceramic caused less amount of wear than glazed IPS ceramic. Antagonist wear of different surface polishes of zirconia ceramics was conducted by J. Park et al. [18] who also concluded that surface roughness and wear was more after staining and glazing than polished. Similarly, Albashaireh et al. [19] conducted a study to compare the wear behavior of ceramics and concluded that zirconia specimens demonstrated the lowest volumetric loss than other ceramics. However, the findings of Monasky and Taylor [14], Wiley [5], and Jagger and Harrison [16] all concluded that there is no notable statistical disparity in the mean surface roughness between the ultimate polished surface and the initial autoglazed surface of ceramics.

However, when the main groups were compared individually it was observed that there were no significant results. When the autoglazed groups of IPS Empress and Zirconia were compared it was observed that there were no significant results obtained. Similarly, when the overglazed groups and polished groups of IPS Empress and Zirconia were compared it was observed that there were no significant results obtained. The results for weight loss after 25,000 cycles for ceramic surfaces that had been overglazed were just a little bit greater than those for ceramic surfaces that had been autoglazed. This was in line with the study by Tholt et al. [20] who concluded that the wear caused by polished and glazed surfaces is negligible and of no clinical consequence. Zandparsa et al. [21] also compared the wear of advanced ceramics like Zirconia and IPS E-max and concluded that there was no linear and volumetric reduction of tooth samples. Within the confines, the findings reached that there was less enamel wear with the polished groups for both IPS Empress and Zirconia and the overglazed group showed the maximum amount of wear when the two groups were considered. In another study, it was found that reduced enamel wear with polished crowns, as evidenced by the mean antagonistic enamel wear of teeth opposing polished and glazed zirconia [22].

Limitations

The study has some limitations, the sample size was small, and due to economic constraints larger sample size could not be tested. In vitro studies could only check wear by sliding the two surfaces in a back and fro motion and cannot accurately stimulate the wear as it happens in the oral cavity. Furthermore, the dynamics of ingested food, alterations in pH levels, and fluctuations in temperature, which hold paramount relevance in a real-world setting, were not duly considered within the study's parameters. The awareness of these constraints contributes to a well-rounded understanding of the study's scope and its potential implications for the broader field of dental research.

## Conclusions

The study highlights the importance of considering surface finishes in ceramic restorations to minimize enamel wear and promote long-term oral health. Polishing of ceramic surfaces could offer a favorable alternative to glazing in terms of reducing wear on opposing teeth. These findings contribute to the ongoing research on improving dental materials and techniques to ensure optimal aesthetic and functional outcomes in restorative dentistry. Further investigations with larger sample sizes and longer wear cycles could provide additional insights into the behavior of different ceramic materials and surface finishes in clinical settings.
